# Inhibitory Effect of a Human MicroRNA, miR-6133-5p, on the Fibrotic Activity of Hepatic Stellate Cells in Culture

**DOI:** 10.3390/ijms21197251

**Published:** 2020-10-01

**Authors:** Susumu Hamada-Tsutsumi, Masaya Onishi, Kentaro Matsuura, Masanori Isogawa, Keigo Kawashima, Yusuke Sato, Yasuhito Tanaka

**Affiliations:** 1 Department of Virology and Liver Unit, Nagoya City University Graduate School of Medical Sciences, Nagoya 467-8601, Japan; tsutsumi@med.nagoya-cu.ac.jp (S.H.-T.); om19840905@gmail.com (M.O.); misogawa@med.nagoya-cu.ac.jp (M.I.); keepitgood0110@yahoo.co.jp (K.K.); 2 Department of Gastroenterology and Metabolism, Nagoya City University Graduate School of Medical Sciences, Nagoya 467-8601, Japan; matsuura@med.nagoya-cu.ac.jp; 3 Laboratory of Innovative Nanomedicine, Faculty of Pharmaceutical Sciences, Hokkaido University, Sapporo 060-0812, Japan; y_sato@pharm.hokudai.ac.jp; 4 Department of Gastroenterology and Hepatology, Faculty of Life Sciences, Kumamoto University, Kumamoto 860-8556, Japan

**Keywords:** liver fibrosis, hepatic stellate cells, TGF-β, JNK signaling pathway

## Abstract

Background: We recently identified 39 human microRNAs, which effectively suppress hepatitis B virus (HBV) replication in hepatocytes. Chronic HBV infection often results in active, hepatitis-related liver fibrosis; hence, we assessed whether any of these microRNAs have anti-fibrotic potential and predicted that miR-6133-5p may target several fibrosis-related genes. Methods: The hepatic stellate cell line LX-2 was transfected with an miR-6133-5p mimic and subsequently treated with Transforming growth factor (TGF)-β. The mRNA and protein products of the *COL1A1* gene, encoding collagen, and the *ACTA2* gene, an activation marker of hepatic stellate cells, were quantified. Results: The expression of *COL1A1* and *ACTA2* was markedly reduced in LX-2 cells treated with miR-6133-5p. Interestingly, phosphorylation of c-Jun N-terminal kinase (JNK) was also significantly decreased by miR-6133-5p treatment. The expression of several predicted target genes of miR-6133-5p, including *TGFBR2* (which encodes Transforming Growth Factor Beta Receptor 2) and *FGFR1* (which encodes Fibroblast Growth Factor Receptor 1), was also reduced in miR-6133-5p-treated cells. The knockdown of *TGFBR2* by the corresponding small interfering RNA greatly suppressed the expression of *COL1A1* and *ACTA2*. Treatment with the JNK inhibitor, SP600125, also suppressed *COL1A1* and *ACTA2* expression, indicating that TGFBR2 and JNK mediate the anti-fibrotic effect of miR-6133-5p. The downregulation of *FGFR1* may result in a decrease of phosphorylated Akt, ERK (extracellular signal-regulated kinase), and JNK. Conclusion: miR-6133-5p has a strong anti-fibrotic effect, mediated by inactivation of TGFBR2, Akt, and JNK.

## 1. Introduction

The progression of liver fibrosis often leads to fatal outcomes, such as the development of cirrhosis and hepatocellular carcinoma. Infections with viruses such as the hepatitis B virus (HBV) and hepatitis C virus are the major causes of liver fibrosis and contributed to around 50% of cirrhosis and hepatocellular carcinoma cases in 2017 [1]. Approximately 250 million people worldwide were infected with hepatitis viruses, resulting in nearly 1.4 million deaths in 2016, which is more than those caused by human immunodeficiency virus infection or tuberculosis [2]. Therefore, the development of novel treatment options to prevent the progression of liver fibrosis is important for reducing risks to health.

Hepatic stellate cells (HSCs) are major producers of extracellular matrix proteins, such as collagen fibers, during the development of fibrosis. HSCs are activated by fibrogenic cytokines, such as Transforming growth factor (TGF)-β, angiotensin II, and leptin, induced by liver injury [3]. Once activated, HSCs proliferate and differentiate into myofibroblasts and start to produce α-smooth muscle actin (α-SMA). Although extensive efforts have revealed that various signaling molecules such as Akt and c-Jun N-terminal kinase (JNK) control the activation and fibrogenesis of HSCs [4,5,6,7,8], the molecular processes involved in HSC activation are not entirely understood [9].

MicroRNAs (miRNAs) are small non-coding RNAs, 21–25 nucleotides in length, encoded in the human genome. Each miRNA targets hundreds of mRNAs and downregulates them post-transcriptionally by base pairing with their 3′-untranslated regions (3′-UTRs) [10,11]. Extensive studies have revealed that miRNAs regulate various biological and cellular processes, including proliferation, differentiation, cell behavior, and cancer development [12,13,14,15,16,17]. The involvement of miRNAs in fibrosis of the liver and other organs also has been reported [18,19,20,21,22].

Recently, by screening a human miRNA mimic library, we identified 39 miRNAs that effectively suppress HBV replication [23]. A significant portion of chronically HBV-infected patients suffer from progression of liver fibrosis toward cirrhosis [24]. Hence, we investigated whether any of these miRNAs have additional effects related to fibrosis and found that one of them, miR-6133-5p, potentially targets several fibrosis-related genes. Interestingly, miR-6133 greatly suppressed not only the *COL1A1* gene that encodes the α-chain of collagen type I (collagen Iα1), the major component of fibrous tissue in the liver, but also the *ACTA2* gene, which encodes α-SMA, indicating its anti-fibrotic potential. In the present study, we explored the molecular mechanisms by which miR-6133-5p suppresses the fibrotic activity of hepatic stellate cells.

## 2. Results

### 2.1. MiR-6133-5p Suppresses the Synthesis of α-Chain of Collagen Type I and α-Smooth Muscle Actin in LX-2 Cells

**Figure 1:**
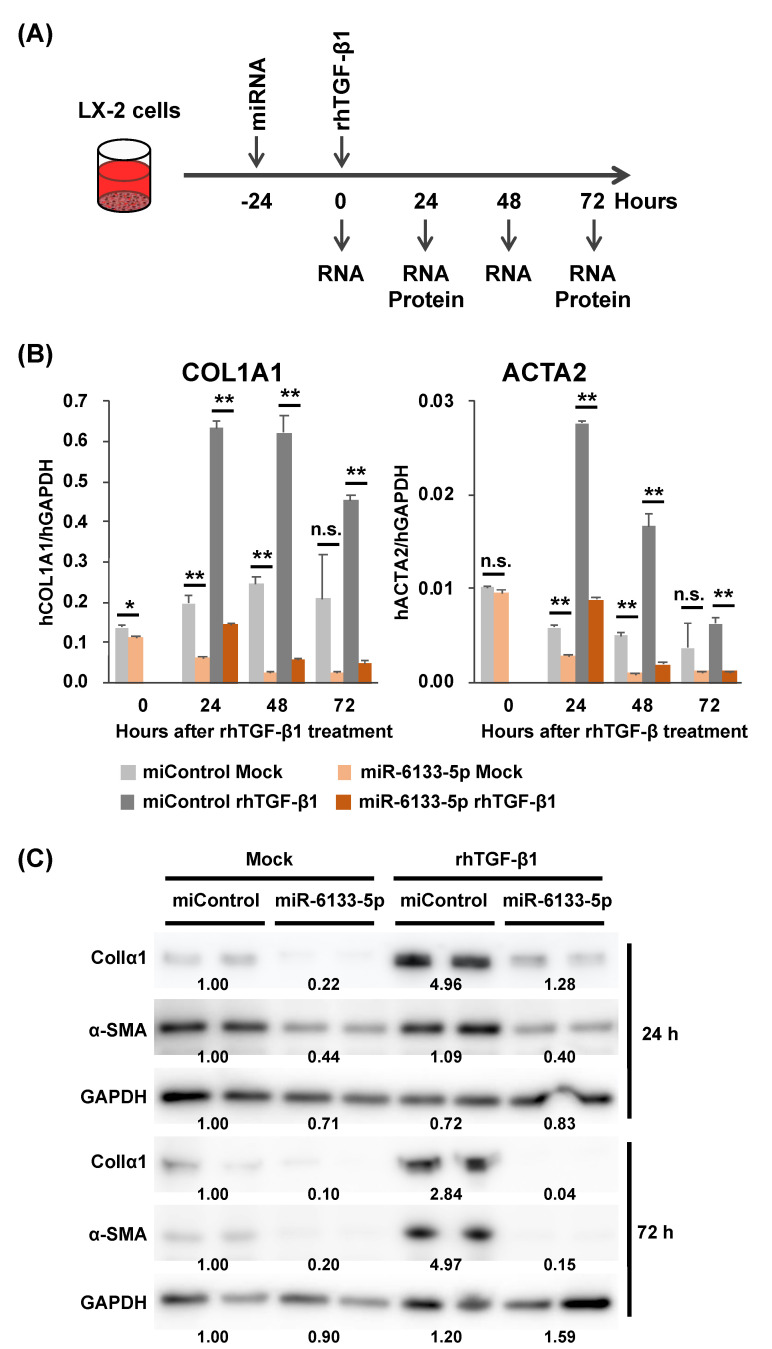
The impact of miR-6133 on the anti-fibrotic activity of LX-2 cells. (**A**) LX-2 cells were transfected with a miR-6133-5p mimic or a negative control microRNA mimic (miControl) at a final concentration of 20 nM, 24 h before treatment with recombinant human transforming growth factor β1 (rhTGF-β1, 5 ng/mL). Total RNA and proteins were extracted from the cells collected at the time points indicated. (**B**) The expression of the *COL1A1* gene encoding collagen Iα1, and the *ACTA2* gene encoding α-smooth muscle actin was determined by RT-qPCR. (**C**) The amounts of α-chain of collagen type I (collagen Iα1) and α-smooth muscle actin (α-SMA) were determined by Western blot analysis. Error bars represent means ± standard deviations (*n* = 3). * *p* < 0.05; ** *p* < 0.01., n.s. not significant.

To explore the role of miR-6133-5p in HSC functions, we transfected an miR-6133-5p mimic or a negative control miRNA mimic (hereafter referred to as miControl) into a human HSC line, LX-2, 24 h before treatment with 5 ng/mL of recombinant human transforming growth factor β1 (rhTGF-β1), a strong inducer of fibrogenesis. RNA and protein were collected at each time point, as indicated in Figure 1A. As shown in Figure 1B, in the miControl-treated cells, rhTGF-β1 treatment dramatically increased the expression of *COL1A1* and *ACTA2*. Interestingly, the levels of *COL1A1* and *ACTA2* were significantly decreased in the miR-6133-5p-treated cells, irrespective of rhTGF-β1 treatment, indicating that miR-6133 has strong anti-fibrotic property. Western blot analyses showed that the amounts of collagen Iα1 and α-SMA were also decreased in the miR-6133-5p-transfected cells, with or without rhTGF-β1 treatment (Figure 1C). It was noted that the amount of α-SMA protein increased only 72 h after rhTGF-β1 treatment.

**Figure 2:**
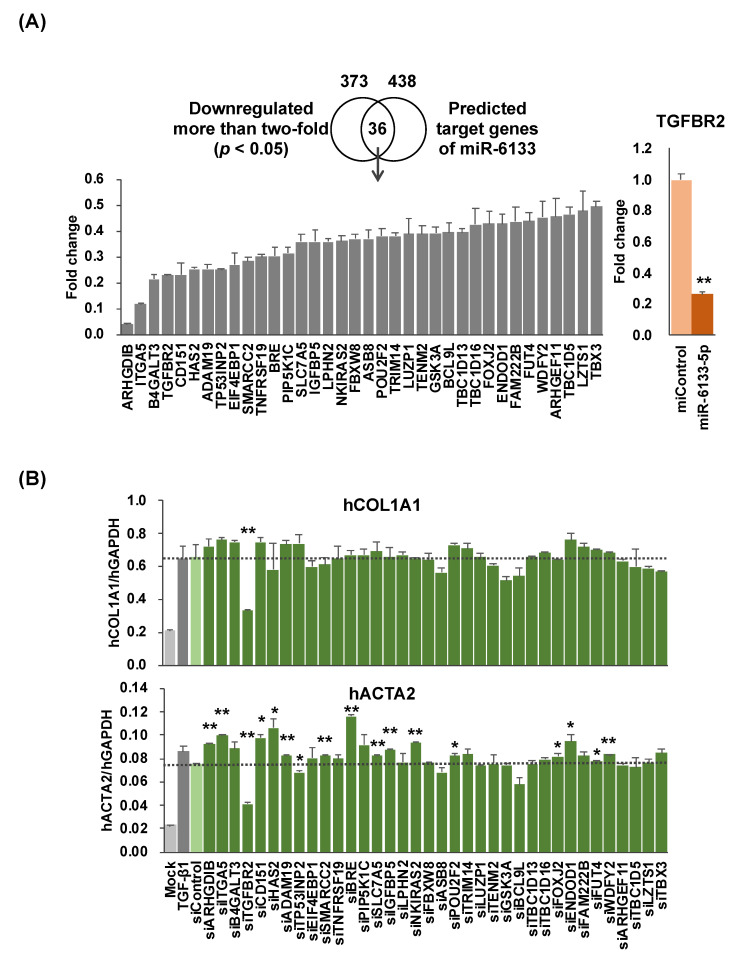
The impact of miR-6133 target genes on the anti-fibrotic activity of LX-2 cells. (**A**) RNAseq analysis revealed that 373 genes were downregulated in miR-6133-treated LX-2 cells compared with those treated with miControl, with statistical significance (*p* < 0.05). Thirty-six of these genes were also among 438 putative miR-6133-5p target genes predicted by TargetScanHuman v7.2. The levels of *TGFBR2* (which encodes Transforming Growth Factor Beta Receptor 2) in the miR-6133-treated and miControl-treated LX-2 cells (*n* = 3) were determined by RT-qPCR. (**B**) LX-2 cells were transfected with small interfering RNAs (siRNAs) corresponding to each of the 36 genes or a non-targeting control siRNA (siControl) at a final concentration of 20 nM, 24 h before treatment with rhTGF-β1 (5 ng/mL). The expression of *COL1A1* and *ACTA2* was determined by RT-qPCR. Fold change was calculated as the ratio over the expression in the siControl-treated sample (indicated by a dotted line). Error bars represent means ± standard deviations (*n* = 3). * *p* < 0.05; ** *p* < 0.01.

**Figure 3:**
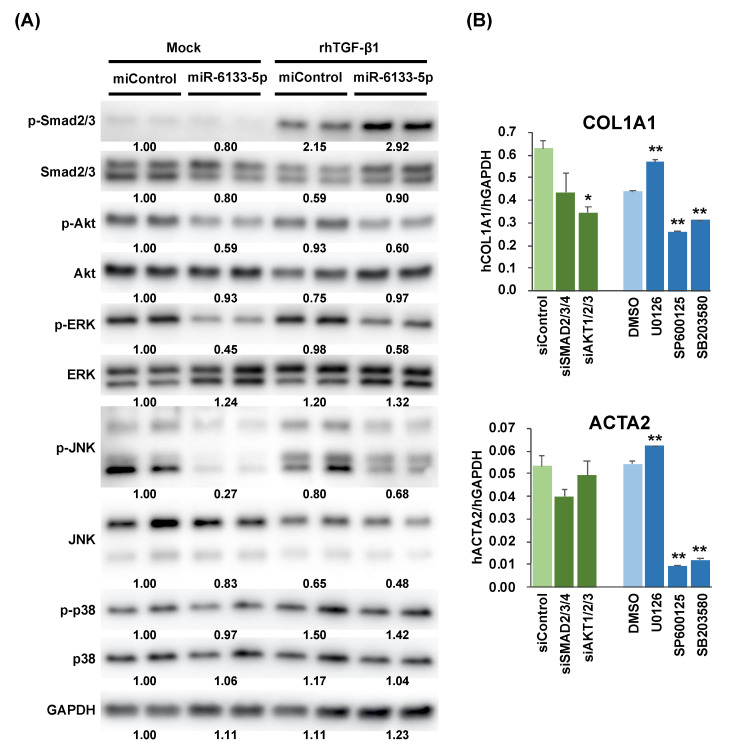
The effect of miR-6133 on the activation of intracellular signaling pathways. (**A**) The amounts of phosphorylated-Smad2/3 (p-Smad2/3), total Smad2/3, p-Akt, Akt, p-ERK (extracellular signal-regulated kinase), ERK, p-JNK (c-Jun N-terminal kinase), JNK, p-p38, p38, and GAPDH (glyceraldehyde-3-phosphate dehydrogenase) were determined by western blot analysis, using the LX-2 extracts collected 24 h after rhTGF-β1 treatment. (**B**) LX-2 cells were treated with a mixture of siRNAs corresponding to the *SMAD2*, *SMAD3*, and *SMAD4* genes (siSMAD2/3/4), a mixture of siRNAs corresponding to the *AKT1*, *AKT2*, and *AKT3* genes (siAKT1/2/3), or siControl, at a final concentration of 30 nM (10 nM for individual siRNA), 24 h before treatment with rhTGF-β1 (5 ng/mL). LX-2 cells were also treated side by side with an MEK (MAPK/ERK kinase) inhibitor (U0126), a JNK inhibitor (SP600125), a p38 inhibitor (SB203580), or DMSO (dimethylsulfoxide), 24 h before rhTGF-β1 treatment. Total RNA was extracted from the samples collected 24 h after rhTGF-β1 treatment and the expression of COL1A1 and ACTA2 was determined by RT-qPCR analysis. Error bars represent means ± standard deviations (*n* = 3). * *p* < 0.05; ** *p* < 0.01.

To identify the molecular mechanisms by which miR-6133-5p suppressed *COL1A1* and *ACTA2*, we performed an RNAseq analysis to compare gene expression patterns of the cells transfected with miR-6133-5p with those transfected with miControl, 24 h after rhTGF-β treatment. Several genes (*n* = 373) were downregulated by more than 50%, with statistical significance (Figure 2A). Among them, 36 genes were also found among the putative target genes of miR-6133-5p predicted by TargetScanHuman v7.2 (total 438 genes, http://www.targetscan.org/vert_72/) [11]. We then examined the role of each gene on the expression of *COL1A1* and *ACTA2* by transfecting with the corresponding small interfering RNAs (siRNA). As shown in Figure 2B, only knockdown of the *TGFBR2* gene, which encodes a component of the human TGF-β receptor, downregulated *COL1A1* and *ACTA2* more than 20%, compared with cells treated with a non-targeting control siRNA (siControl), with statistical significance. However, suppression of *COL1A1* and *ACTA2* by miR-6133-5p was also observed in the absence of rhTGF-β. Moreover, miR-6133 did not alter rhTGF-β-induced phosphorylation of Smad2/3, indicating the presence of TGFBR2-Smad independent pathways (Figure 3A).

By analyzing the RNAseq data in terms of gene ontology, we found that several genes encoding extracellular matrix proteins and genes involved in epithelial-to-mesenchymal transition, such as *CTGF* (connective tissue growth factor), *COL1A2* (collagen Iα2), *COL5A3* (collagen Vα3), *LOX* (Lysyl oxidase), *SNAI2* (Snail 2), and *CDH2* (Cadherin 2), were also significantly downregulated in the miR-6133-5p-treated group (Supplementary Tables S1 and S2). These results indicated that miR-6133-5p partially affects the activation and fibrotic function of LX-2 cells.

### 2.2. MiR-6133 Decreased Phosphorylation of Akt, ERK, and JNK

We then examined the impact of miR-6133 on the major cellular signaling pathways mediated by the serine/threonine kinases, Akt, ERK (extracellular signal-regulated kinase), JNK, and p38. Surprisingly, the amounts of phosphorylated forms of Akt, ERK, and JNK, but not p38, were smaller in the miR-6133-treated LX-2 cells than the miControl-treated cells, 24 h after rhTGF-β treatment (Figure 3A). Moreover, these differences were also observed in the mock-treated groups (Figure 3A). To determine whether the inhibition of phosphorylation of any of these kinases affected the expression of *COL1A1* and *ACTA2*, we used siRNAs targeting the *SMAD2*, *SMAD3*, and *SMAD4* genes, which are the central mediators of canonical TGF-β signaling and those targeting the *AKT1*, *AKT2*, and *AKT3* genes; and chemical inhibitors of MEK (MAPK/ERK kinase), JNK, and p38.

As shown in Figure 3B, treatment with SMAD2/3/4 siRNAs slightly decreased *COL1A1* and *ACTA2*, but without statistical significance, indicating that *COL1A1* and *ACTA2* are not solely regulated by the canonical TGF-β-Smad2/3/4 pathway. The knockdown of *AKT1/2/3* decreased the level of *COL1A1*, but not *ACTA2*. Treatment with the MEK inhibitor, U0126, which inhibits phosphorylation of ERK by the upstream kinase MEK, slightly increased the levels of *COL1A1* and *ACTA2*. In contrast, inhibition of JNK and p38 by their corresponding inhibitors (SP600125 and SB203580, respectively) significantly suppressed *COL1A1* and *ACTA2* expression (Figure 3B). Collectively, these results suggested that Akt may partially account for the suppression of *COL1A1* by miR-6133-5p and, similarly, JNK may partially account for the suppression of both *COL1A1* and *ACTA2* by miR-6133-5p.

**Figure 4:**
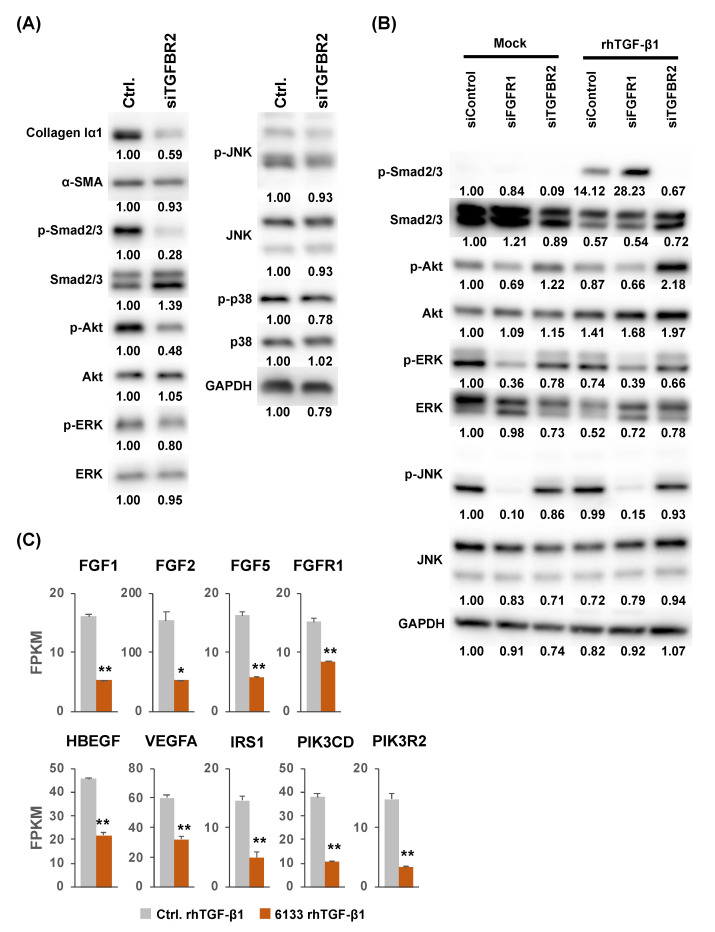
The effect of knockdown of *TGFBR2* and *FGFR1* (which encodes Fibroblast Growth Factor Receptor 1), on the activation of intracellular signaling pathways. (**A**) LX-2 cells were treated with an TGFBR2 siRNA or siControl at a final concentration of 20 nM, 24 h before treatment with rhTGF-β1 (5 ng/mL). Protein extracts were prepared from the samples collected 24 h after rhTGF-β1 treatment and subjected to Western blot analysis. (**B**) LX-2 cells were treated with an FGFR1 siRNA, TGFBR2 siRNA, or siControl at a final concentration of 20 nM, 24 h before treatment with rhTGF-β1. The lysates were analyzed by Western blot analysis. (**C**) Values of fragments per kilobase of exon per million reads mapped (FPKM) for *FGF1*, *FGF2*, *FGF5*, *FGFR1*, *HBEGF*, *VEGFA*, *IRS1*, *PIK3CD*, and *PIK3R2* genes, deduced from the RNAseq analysis comparing samples treated with miR-6133-5p and miControl. Error bars represent means ± standard deviations (*n* = 3). * *p* < 0.05; ** *p* < 0.01.

We next investigated the relationship between the suppression of the *TGFBR2* gene and that of Akt, ERK, and JNK phosphorylation using an siRNA targeting the *TGFBR2* gene (siTGFBR2). As shown in Figure 4A, the amount of collagen Iα1 was greatly decreased and that of α-SMA was slightly decreased in the LX-2 cells treated with siTGFBR2 compared with the siControl-treated cells. While the amount of phosphorylated forms of Smad2/3 and Akt was also significantly decreased by siTGFBR2, it had no impact on the amounts of phosphorylated ERK, JNK, and p38 (Figure 4A). These results indicated that the suppression of JNK by miR-6133 is independent of TGFBR2.

### 2.3. Possible Involvement of the Fibroblast Growth Factor Receptor 1 (FGFR1) Gene in the Suppression of JNK Phosphorylation by MiR-6133-5p

Next, to further elucidate the mechanism by which miR-6133-5p suppressed the phosphorylation of Akt, ERK, and JNK, we performed gene set enrichment analysis (GSEA) and constructed miRNA-mRNA networks from RNAseq data. By analyzing GSEA of gene ontology (GO) gene sets in the miR-6133-5p-treated LX-2 cells, we found that a group of genes annotated as ‘extracellular matrix organization’ were significantly downregulated in the miR-6133-5p-treated cells (Supplementary Figure S1A,B). From the putative miR-6133-target genes predicted by TargetScanHuman v7.2 and experimentally validated miR-6133-5p target genes listed in miRTarBase v8.0 (http://mirtarbase.cuhk.edu.cn/php/index.php) [25], we selected 98 genes whose expression was significantly lower in the miR-6133-5p-treated LX-2 cells (fold change in log2 ratio > 0.8, *p* < 0.05, fold discovery rate < 0.01; Supplementary Figure S1C). Among them, we found the *FGFR1* gene annotated in ‘extracellular matrix organization’, which is known to transmit a signal to the PI3K-Akt and ERK/JNK/p38 signaling pathways upon activation by its ligands, fibroblast growth factors [26]. As shown in Figure 4B, knockdown of *FGFR1* by the corresponding siRNA significantly decreased the phosphorylated form of JNK. It also decreased phosphorylated forms of Akt and ERK to some extent. These results suggested that the anti-fibrotic function of miR-6133-5p may partially be mediated by the FGFR-Akt/ERK/JNK axis. In contrast, knockdown of *FGFR1* increased the amount of phosphorylated form of Smad2/3 (Figure 4B), indicating that FGFR1 acts inhibitory to the TGF-β pathway, including Smad2/3, as reported by Li et al. [27]. Interestingly, the levels of expression of genes encoding FGFR ligands (*FGF1*, *FGF2*, and *FGF5*) were also reduced in the miR-6133-5p-treated cells. The level of genes involved in other growth factor signaling pathways, such as *HBEGF* (the epidermal growth factor signaling pathway), *VEGFA* (the vascular endothelial growth factor signaling pathway), and *IRS1*, *PIK3CD*, *PIK3R2* (the IGF-PI3K signaling pathway), were also significantly decreased (Figure 4C).

## 3. Discussion

**Figure 5:**
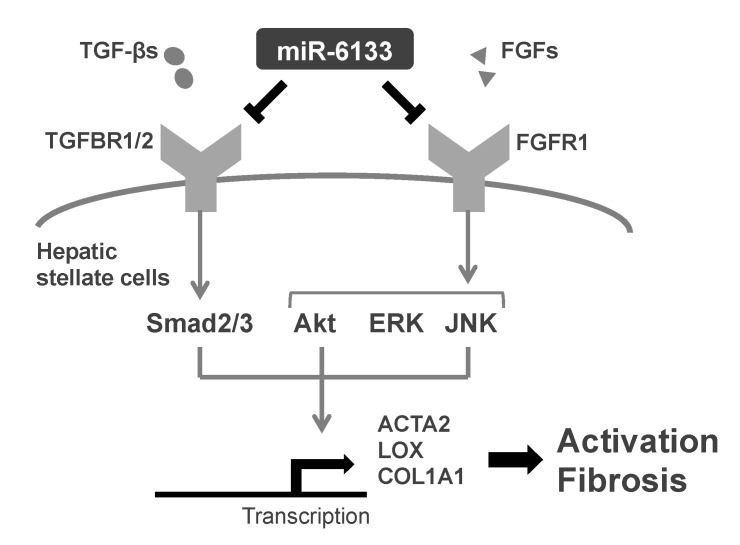
Schema of anti-fibrotic mechanisms via miR-6133-5p. TGF-β signaling mediated by the receptor TGFBR2 and its receivers, Smad2/3, control, *COL1A1*, and *ACTA2*. miR-6133-5p directly suppresses the expression of *TGFBR2*. miR-6133-5p decreases phosphorylation (activation) of Akt, ERK, and JNK, possibly by targeting *FGFR1* which results in the suppression of FGF/FGFR axis. Akt and JNK regulate *COL1A1* and *ACTA2* expression.

Recently, we reported that the human miRNA, miR-6133-5p, has strong antiviral activity against HBV replication [23]. In this study, we found that miR-6133-5p effectively suppressed *COL1A1* and *ACTA2*—the main component of fibrous tissue in the liver and a representative marker of HSC activation, respectively. An RNAseq analysis also revealed that several genes encoding other extracellular matrix proteins and those involved in epithelial-to-mesenchymal transition were significantly downregulated in the miR-6133-5p-treated cells, suggesting that miR-6133-5p has strong, but partial, anti-fibrotic property when introduced in HSCs. Functional analyses revealed that siRNA-mediated knockdown of *TGFBR2* and *AKT1/2/3*, and inhibition of JNK by an appropriate chemical, suppressed *COL1A1* and *ACTA2* expression, suggesting that the anti-fibrotic effects of miR-6133-5p may be mediated by TGFBR2, Akt, and JNK (Figure 5).

The decrease of phosphorylated Akt, ERK, and JNK in the miR-6133-treated LX-2 cells could be due to the downregulation of *FGFR1*, a target gene of miR-6133-5p. Several reports have shown that Akt is involved in collagen synthesis and the activation of HSCs [4,5]. JNK has also been reported to regulate collagen synthesis and the activation of HSCs [6,7,8]. A chemical compound, GS-444217, that specifically inhibit ASK1 (Apoptosis signal-regulating kinase 1), a protein kinase upstream of JNK and p38, has been reported to reduce liver fibrosis in a mouse model with a *Nlrp3* (NLR family pyrin domain containing 3) loss-of-function mutation [28]. These findings, together with our present study, indicate that signaling pathways including Akt and JNK are therapeutic targets for the control of liver fibrosis.

The roles of endogenous miR-6133-5p in humans are largely unknown. miR-6133-5p is expressed in almost all tissues, including the liver. One report revealed that the amount of urinary exosomal miR-6133-5p was increased in type II diabetic nephropathy patients [29]. On the other hand, we found that the level of miR-6133-5p was not altered in the rhTGF-β1-treated LX-2 cells, compared with the control (data not shown), indicating that endogenous level of miR-6133 have no impact on the fibrogenic function of HSCs.

miR-6133-5p is found in the genome of several primates. Although it is important to determine whether miR-6133-5p effectively ameliorates liver fibrosis in vivo, at present, we could not employ physiologically relevant small animal models for evaluating the effect of primate-restricted miRNA such as miR-6133-5p on liver fibrosis in vivo. Chimeric mice, with liver repopulated with human hepatocytes, were frequently used to study HBV replication in vivo [30]. The development of similar experimental animal models such as small animals harboring human HSCs in the liver would help to examine the effect of reagents on liver fibrosis in future studies.

Some issues remain to be addressed. While the expression of *ACTA2* was induced by rhTGF-β1 and peaked 24 h after treatment, the increase of α-SMA protein became obvious only 72 h after rhTGF-β1 treatment (Figure 1B,C). Similarly, while knockdown of *TGFBR2* greatly suppressed *ACTA2* expression 24 h after rhTGF-β1 treatment, the amount of α-SMA protein was not decreased so much (Figure 2B and Figure 4A). This could be due to the balance between the efficiency of translation and the degradation of α-SMA protein, which may cause a delay in the outcome of the increase/decrease of *ACTA2* mRNA and changes in the amount of its protein product.

On the other hand, knockdown of *TGFBR2* by the corresponding siRNA greatly decreased the phosphorylated form of Smad2/3. However, although miR-6133-5p treatment also suppressed *TGFBR2* effectively, it had no impact on the amount of phosphorylated Smad2/3. The amount of phosphorylated Smad2/3 was increased by the knockdown of *FGFR1* (Figure 4B); hence, it is possible that the downregulation of *FGFR1* by miR-6133 may partially cancel the suppressive effect by the downregulation of *TGFBR2* in terms of the level of phosphorylation of Smad2/3.

Our results showed that the inhibition of p38 by its inhibitor, SB203580, also effectively suppressed *COL1A1* and *ACTA2* expression (Figure 3B). On the other hand, miR-6133-5p had no impact on the phosphorylation of p38 in LX-2. JNK and p38 are differently regulated by upstream kinases (MKK4 (mitogen-activated protein kinase kinase 4)/MKK7 and MKK3/MKK6, respectively); hence, miR-6133-5p could selectively inhibit JNK without affecting p38 and it is sufficient for the suppression of *COL1A1* and *ACTA2* in LX-2 cells.

A comprehensive transcriptome analysis covering many time points is needed in a future study to dissect the effect of miR-6133-5p from the immediate-early suppression of direct target genes, middle-stage changes of signaling pathways, and late-stage changes, such as the downregulation of *COL1A1* and *ACTA2*. The role of miR-6133-5p in the fibrosis of other organs or tissues has also not been documented; further studies will be required to examine the effect of miR-6133-5p in various fibroblast cells of different organ origins.

In conclusion, we found that miR-6133-5p has strong anti-fibrotic effect which could be mediated by inactivation of TGFBR2, Akt, and JNK.

## 4. Materials and Methods

### 4.1. Cell Culture and Transfection

LX-2 cells were cultured in Dulbecco’s modified Eagle’s medium (Thermo Fisher Scientific, Waltham, MA, USA) supplemented with 1% antibiotic antimycotic solution and 2% heat-inactivated fetal bovine serum (Thermo Fisher Scientific). MiRIDIAN MicroRNA miR-6133-5p Mimic and MiRIDIAN MicroRNA Mimic Negative Control #1 (miControl) were purchased from Horizon Discovery Group plc. (Cambridge, UK). ON-TARGETplus siRNA SMARTpools targeting *SMAD2*, *SMAD3*, *SMAD4*, *AKT1*, *AKT2*, *AKT3*, and *FGFR1*; and ON-TARGETplus Non-targeting Pool (siControl) were purchased from Horizon Discovery Group plc. Each SMARTpool contains four independent siRNA molecules to ensure efficient gene knockdown. Silencer Select siRNA reagents corresponding to 36 predicted target genes of miR-6133-5p and Silencer Select negative control siRNA were purchased from Thermo Fisher Scientific. Two independent Silencer Select siRNA molecules for each gene were mixed equally and used in the following assay to ensure efficient gene knockdown. The sequence information of the siRNAs used in this study was shown in Supplementary Table S4.

miRNAs or siRNAs were introduced into LX-2 cells at a final concentration of 20 nmol/L using Lipofectamine RNAiMAX Reagent (Thermo Fisher Scientific), according to the manufacturer’s instructions. Twenty-four hours after transfection, the cells were further cultured with medium containing 5 ng/mL of rhTGF-β1. The cells were then harvested and subjected to RNA and protein extraction at the time points indicated in Figure 1A.

### 4.2. Gene Expression Analysis by RT-qPCR

Total RNA was extracted using ISOGEN (Nippon Gene, Toyama, Japan). Gene expression was determined by RT-qPCR using StepOne Plus (Thermo Fisher Scientific). The primer–probe sets for RT-qPCR analysis of human *COL1A1*, *ACTA2*, *TGFBR2*, *FGFR1*, and *GAPDH* genes were purchased from Thermo Fisher Scientific.

### 4.3. Quantification of Protein

Cell lysates were prepared using a lysis buffer containing 1% Nonidet P40, 150 mM sodium chloride, and 50 mM Tris-Cl buffer (pH 7.4). A cOmplete Mini EDTA-free tablet and a PhosSTOP tablet (Roche diagnostics, Basel, Switzerland) were added to each 10 mL of the lysis buffer immediately before use. Western blot analyses were performed by a routine procedure using the primary antibodies listed below with the species, target, company, catalogue number, and dilution: mouse anti-collagen Iα1 (sc-293182, Santa Cruz, Dallas, TX, USA, 1:1000); rabbit anti-α-SMA (GTX100034, GeneTex, Irvine, CA, USA, 1:1000); rabbit anti-phospho-Smad2/3 (#8828, Cell Signaling Technology, Danvers, MA, USA, 1:2000); rabbit anti-Smad2/3 (#8685, Cell Signaling Technology, 1:2000); rabbit anti-phospho-Akt (#9271, Cell Signaling Technology, 1:2000); rabbit anti-Akt (#9272, Cell Signaling Technology, 1:2000); mouse anti-phospho-ERK (#9106, Cell Signaling Technology, 1:2000); rabbit anti-ERK (#9102, Cell Signaling Technology, 1:2000); rabbit anti-phospho-JNK (#9251, Cell Signaling Technology, 1:2000); rabbit anti-JNK (#9252, Cell Signaling Technology, 1:2000); rabbit anti-phospho-p38 (#9211, Cell Signaling Technology, 1:2000); rabbit anti-p38 (#9212, Cell Signaling Technology, 1:2000); mouse anti-GAPDH (ab8245, Abcam, Cambridge, UK, 1:10,000). The intensity of the bands was calculated using ImageJ v1.8.0 (https://imagej.nih.gov/ij/index.html).

### 4.4. RNAseq Analysis

Total RNA samples collected from miR-6133-5p- and miControl-treated LX-2 cells (*n* = 2) 24 h after rhTGF-β1 treatment were subjected to an RNAseq analysis. PolyA + RNA was extracted, fragmented, and reverse-transcribed to yield a single-stranded cDNA mixture. Double-stranded DNA was then synthesized using the cDNA mixture as a template. The ends of the product were blunted, phosphorylated, followed by addition of 3′-deoxyadenosine, and ligated with adapter DNA fragments containing an index sequence unique to each sample. After amplification by PCR, the resultant sequencing libraries were subjected to pair-end sequencing (sequence length = 150 bases) using NovaSeq 6000, NovaSeq 6000 S4 Reagent Kit, and NovaSeq Xp 4-Lane Kit (Illumina Inc., San Diego, CA, USA). The reads were mapped and annotated using GeneData Profiler Genome v11.0.4a (GeneData, Basel, Switzerland) and STAR v2.5.3a (https://github.com/alexdobin/STAR). *Homo sapiens* genome assembly GRCh37 (hg19) was used as the reference. The number of reads and the percentage of mapped reads for each sample are shown in Supplementary Table S3. Read counts underwent the trimmed mean of M values (TMM) normalization and log2 computes counts per million (CPM) transformation using the edgeR software v3.30.3 [31]. Differences in gene expression between miR-6133-5p and miControl were tested by a quasi-likelihood test function (glmQLFit). We set a false-discovery rate (FDR) threshold of 0.01 to correct for multiple testing and set a log-fold change (Log2FC) threshold of 0.8. The RNAseq data were deposited in the Gene Expression Omnibus database (accession number: GSE158478, https://www.ncbi.nlm.nih.gov/geo/).

To functionally characterize miR-6133-5p, we performed a pathway analysis using the GSVA R package (https://www.bioconductor.org/packages/release/bioc/html/GSVA.html) [32]. The gene sets used were the Kyoto Encyclopedia of Genes and Genomes (KEGG) pathway and all GO gene sets from the Broad Institute’s Molecular Signatures Database (MSigDB) v7.1. The top differentially enriched pathways were yielded along with *p*-values adjusted for multiple testing correction using the Benjamini–Hochberg FDR controlling procedure. Cytoscape software v3.6.2 [33] was employed to construct the miRNA–mRNA gene network. All data were analyzed in R (http://www.r-project.org/).

### 4.5. Statistical Analysis

The student’s *t*-test was performed using Microsoft Excel. Data are depicted as the mean ± standard deviation, and *p*-values < 0.05 were considered significant: * *p* < 0.05, ** *p* < 0.01.

## Supplementary Materials

Supplementary materials can be found at https://www.mdpi.com/1422-0067/21/19/7251/s1. Table S1: Downregulated genes listed in the gene set ‘Extracellular matrix’; Table S2: Downregulated genes listed in the gene set ‘Hallmark epithelial mesenchymal transition’; Table S3: The RNAseq Results; Table S4: Sequence information of the siRNAs used in this study; Figure S1: Characterization of genes controlled by miR-6133-5p.

## Abbreviations

HBV	hepatitis B virus
HSCs	hepatic stellate cells
TGF-β	transforming growth factor β
α-SMA	α-smooth muscle actin
miRNA	microRNA
3′-UTRs	3′-untranslated regions
ERK	extracellular signal-regulated kinase
JNK	c-Jun N-terminal kinase
FGFR1	fibroblast growth factor 1
siRNA	small interfering RNA
GSEA	gene set enrichment analysis
GO	gene ontology
