# Supplementary material for: Inhibitory Effect of a Human MicroRNA, miR-6133-5p, on the Fibrotic Activity of Hepatic Stellate Cells in Culture
Source: Int J Mol Sci. 2020 Oct 1;21(19):7251. doi: 10.3390/ijms21197251 (PMC7583928; doi:10.3390/ijms21197251)
Supplement: Supplementary file 1 [file ijms-21-07251-s001.pdf]

International Journal of
Molecular Sciences

MDPI

Supplementary

Inhibitory effect of a human microRNA, miR-6133-5p, on the fibrotic activity of hepatic stellate cells in culture

Susumu Hamada-Tsutsumi [1], Masaya Onishi [1], Kentaro Matsuura [2], Masanori Isogawa [1], Keigo Kawashima [1], Yusuke Sato [3] and Yasuhito Tanaka [1,4,*]

[1] Department of Virology and Liver unit, Nagoya City University Graduate School of Medical Sciences, Nagoya 467-8601, Japan; tsutsumi@med.nagoya-cu.ac.jp
[2] Department of Gastroenterology and Metabolism, Nagoya City University Graduate School of Medical Sciences, Nagoya 467-8601, Japan; matsuura@med.nagoya-cu.ac.jp
[3] Laboratory of Innovative Nanomedicine, Faculty of Pharmaceutical Sciences, Hokkaido University, Sapporo 060-0812, Japan; y_sato@pharm.hokudai.ac.jp
[4] Department of Gastroenterology and Hepatology, Faculty of Life Sciences, Kumamoto University, Kumamoto, 860-8556, Japan; ytanaka@kumamoto-u.ac.jp
* Correspondence: ytanaka@kumamoto-u.ac.jp: Tel.: +81-96-373-5146

Table of contents

Supplementary Table S1

Supplementary Table S2

Supplementary Table S3

Supplementary Table S4

Supplementary Figure S1

Table 1. Downregulated genes listed in the gene set ‘Extracellular matrix’.

Gene symbol	Gene name	Fold change (log2)	P-value
CTGF	connective tissue growth factor	-2.154	0.005
FBLN5	fibulin 5	-1.670	0.000
LAMC1	laminin, gamma 1 (formerly LAMB2)	-1.385	0.029
SGCD	sarcoglycan, delta (35kDa dystrophin-associated glycoprotein)	-1.367	0.039
COL5A3	collagen, type V, alpha 3	-1.315	0.002
COL1A2	collagen, type I, alpha 2	-0.906	0.003
LTBP2	latent transforming growth factor beta binding protein 2	-0.760	0.031

Genes whose expression was downregulated more than 0.7 log2 with statistical significance (P > 0.05) were selected from the gene set ‘Extracellular matrix’ (Molecular Signature Database version 7.1, https://www.gsea-msigdb.org/gsea/msigdb).

Table 2. Downregulated genes listed in the gene set ‘Hallmark epithelial mesenchymal transition’.

Gene symbol	Gene name	Fold change (log2)	P-value
CDH6	cadherin 6, type 2, K-cadherin (fetal kidney)	-3.171	0.014
ITGA5	integrin, alpha 5 (fibronectin receptor, alpha polypeptide)	-3.106	0.037
INHBA	inhibin, beta A	-2.333	0.006
PRRX1	paired related homeobox 1	-2.231	0.001
TIMP3	TIMP metallopeptidase inhibitor 3	-2.156	0.002

IGFBP3	insulin-like growth factor binding protein 3	-1.945	0.002
FZD8	frizzled class receptor 8	-1.753	0.002
FBLN5	fibulin 5	-1.670	0.000
LRRC15	leucine rich repeat containing 15	-1.510	0.043
LAMC1	laminin, gamma 1 (formerly LAMB2)	-1.385	0.029
SGCD	sarcoglycan, delta (35kDa dystrophin-associated glycoprotein)	-1.367	0.039
COL5A3	collagen, type V, alpha 3	-1.315	0.002
CALU	calumenin	-1.262	0.002
CDH11	cadherin 11, type 2, OB-cadherin (osteoblast)	-1.225	0.016
ITGB3	integrin, beta 3 (platelet glycoprotein IIIa, antigen CD61)	-1.221	0.013
LAMC2	laminin, gamma 2	-1.146	0.010
LOX	lysyl oxidase	-1.072	0.006
MMP14	matrix metallopeptidase 14 (membrane-inserted)	-1.007	0.018
SNAI2	snail family zinc finger 2	-0.991	0.029
SLIT2	slit homolog 2 (Drosophila)	-0.960	0.017
LOXL2	lysyl oxidase-like 2	-0.945	0.042
SERPINE 1	serpin peptidase inhibitor, clade E (nexin, plasminogen activator inhibitor type 1), member 1	-0.917	0.021
VEGFA	vascular endothelial growth factor A	-0.907	0.006
COL1A2	collagen, type I, alpha 2	-0.906	0.003
WIPF1	WAS/WASL interacting protein family, member 1	-0.882	0.027
TAGLN	transgelin	-0.877	0.030
NNMT	nicotinamide N-methyltransferase	-0.763	0.043
EDIL3	EGF-like repeats and discoidin I-like domains 3	-0.740	0.033
ITGAV	integrin, alpha V	-0.716	0.011
CDH2	cadherin 2, type 1, N-cadherin (neuronal)	-0.709	0.001
SFRP1	secreted frizzled-related protein 1	-0.707	0.004
OXTR	oxytocin receptor	-0.705	0.041

Genes whose expression was downregulated more than 0.7 log2 with statistical significance ($P > 0.05$) were selected from the gene set 'Hallmark epithelial mesenchymal transition' (Molecular Signature Database version 7.1, https://www.gsea-msigdb.org/gsea/msigdb).

Table 3. The RNAseq Results.

Sample ID	Treatment	Total reads	Mapped reads
PR1012_17.a	miControl	64,391,684	97.9%
PR1012_18.a	miControl	71,971,606	98.1%
PR1012_19.a	miR-6133-5p	76,629,170	98.2%
PR1012_20.a	miR-6133-5p	118,633,136	98.0%

Table 4. Sequence information of the siRNAs used in this study.

Gene	Target sequences
SMAD2	GAAUUGAGCCACAGAGUAA
	GGUUUACUCUCCAAUGUUA
	UCAUAAAGCUUCACCAAUC
	ACUAGAAUGUGCACCAUAA
SMAD3	CAACAGGAAUGCAGCAGUG
	GAGUUCGCCUUCAAUAUGA
	GGACGCAGGUUCUCCAAAC
	UUAGAGACAUCAAGUAUGG
SMAD4	GCAAUUGAAAGUUUGGUAA
	CCCACAACCUUUAGACUGA
	GAAUCCAUAUCACUACGAA
	GUACAGAGUUACUACUUAG
AKT1	CAUCACACCACCUGACCAA
	ACAAGGACGGGCACAUUAA

	CAAGGGCACUUUCGGCAAG
	UCACAGCCCUGAAGUACUC
AKT2	ACACAAGGUACUUCGAUGA
	GCAAGGCACGGGCUAAAGU
	GUGAAUACAUCAAGACCUG
	CAUGAAUGACUUCGACUAU
AKT3	GCACACACUCUAACUGAAA
	GAAGAGGGGAGAAUAUAUA
	GUACCGUGAUCUCAAGUUG
	GACAGAUGGCUCAUUCAUA
FGFR1	GCCACACUCUGCACCGCUA
	CCACAGAAUUGGAGGCUAC
	CAAAUGCCCUUCCAGUGGG
	GAAAUUGCAUGCAGUGCCG
ARHGDIB	CCAUGGACCUUACUGGAGA
	GUGGAUAAAGCAACAUUUA
ITGA5	GCAGAGAGAUGAAGAUCUA
	GCAGUGCUAUUCCCAGUAA
B4GALT3	GGACCGACAUUUGACUAUU
	CACUUACUCCUGACCAGUA
TGFBR2	CAACGGUGCAGUCAAGUUU
	CCAAUAUCCUCGUGAAGAA
CD151	GCCUUUUGCUGCGCACCAA
	CCCAACUACUGAGCUGAGA
HAS2	GUAUCUGCAUCAUGCAAAA
	CCUGGGCUAUGCAACAAAA
ADAM19	GCAGCAUGAACUUAUCAUA
	GUUGAUAAGUUUUACCGAU
TP53INP2	CCUUACAUGUCUCACACUA
	CAUUCCCAGUAAUUCCCUA
EIF4EBP1	GAACUCACCUGUGACCAAA
	CGAACCCUUCCUUCCGAAU
SMARCC2	CCUCAACACCUUACACUAA
	GCUACUAUCCUGACAGUUA
TNFRSF19	GGAUUUUAUAGGAAGACGA
	GGAUUCAAAUAGCAGUCAA
BRE	GCACAGGUGUCGUGGAAUA
	CAGGUGUCGUGGAAUAUGA
PIP5K1C	AGACCGUCAUGCACAAGG
	GCGUCGUGGUCAUGAACAA
SLC7A5	UGUCCAAUCUAGAUCCCAA
	ACAGAAAGCCUGAGCUUGA
IGFBP5	AGAAAGCAGUGCAAACCUU
	GCCCAAUUGUGACCGCAAA
LPHN2	CUGCAACAAUGUUACUCGA
	GGCAUAUCUCUUCACUAUA
NKIRAS2	CGUCCUGGUCUAUAGCACA
	GGCUACGUCCUGGUCUAUA
FBXW8	CCAAGUUGCUUUUGGUGUA
	GUGCCUUACCAGACGGUAA
ASB8	CUUCCCACAUGAUAAUGUA
	GGAUGGGUAUAACCGAACA
POU2F2	GAAAUGGACCAGACACUAA
	AGUUACUACCUUAUCCUCA
TRIM14	GAUAAAAACACGCAGCUUA
	GCUAAUGCAGAGUCAAGUA
LUZP1	CUCUGAGCUUUGUAAGUGA
	GUAUCCUUAUAGCUGUAGA

TENM2	GGACUCGAAGGUUCACGAA
	CGUUCGACCUGAUCGCAAA
GSK3A	GGCUUACACGGACAUCAAA
	ACACCAACCCGGGAACAAA
BCL9L	AGAAAUAUGAGGAACCCUU
	GGAGUACUACGAAGAGAAA
TBC1D13	UGCUCAUGCUGAUCCGGGA
	CCUUUUUCUGCUUCACCAA
TBC1D16	GGACAGAUCGGAACAACCA
	GGAGUUUGCUGUACCAGUU
FOXJ2	GGACUCAGCAGGAUACAAU
	GGAAGAAUUCAAUACGGCA
ENDOD1	GGAUGAAGAACGAAUGGUA
	CCGGGACAGUGACAUCAUA
FAM222B	CUAAGAAGGUCGCAAACAA
	ACAACCCACUGACUAUAAA
FUT4	AGUAUUUAAUGAAACCCUA
	GGUCCGCUACUACCACCAA
WDFY2	GGAACUGACAAGGUUAUUA
	GCAUGUCUUUUAACCCGGA
ARHGEF11	GAGAUGAAACGGUCUCGAA
	GCGAAACCCUAUCCUCAAA
TBC1D5	GGGAAGAACUAUUUGUAAA
	GAAUUAAGAGCAUGGUAUA
LZTS1	UCAAGAAGCUCAACCGGUA
	CGGCAAGUCCAGCUCCAAA
TBX3	AAGUGAGACUAUUAGACAA
	CCAUUUAAAGUGAGAUGUU

(A) Differentially Expressed Genes

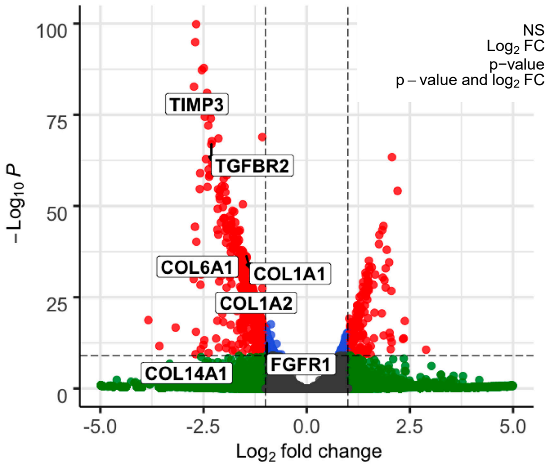

(B) Gene set enrichment analysis

Direction	Pathways	statistic (score)	nGenes	adj.Pval
Down	Blood vessel development	-5.695	498	3.60E-05
Down	Blood vessel morphogenesis	-5.1444	430	0.00037
Down	Mesenchyme development	-4.8552	196	0.0013
Down	Tissue morphogenesis	-4.7191	493	0.0015
Down	Angiogenesis	-4.3769	361	0.0061
Down	Extracellular structure organization	-4.269	273	0.0083
Down	Positive regulation of cell migration	-4.2306	380	0.0083
Down	Mesenchyme morphogenesis	-4.2269	42	0.012
Down	Positive regulation of cell motility	-4.1393	388	0.011
Down	**Extracellular matrix organization**	-4.1249	244	0.011
Down	Mesenchymal cell differentiation	-4.1149	157	0.011

(C) miRNA-mRNA interaction network

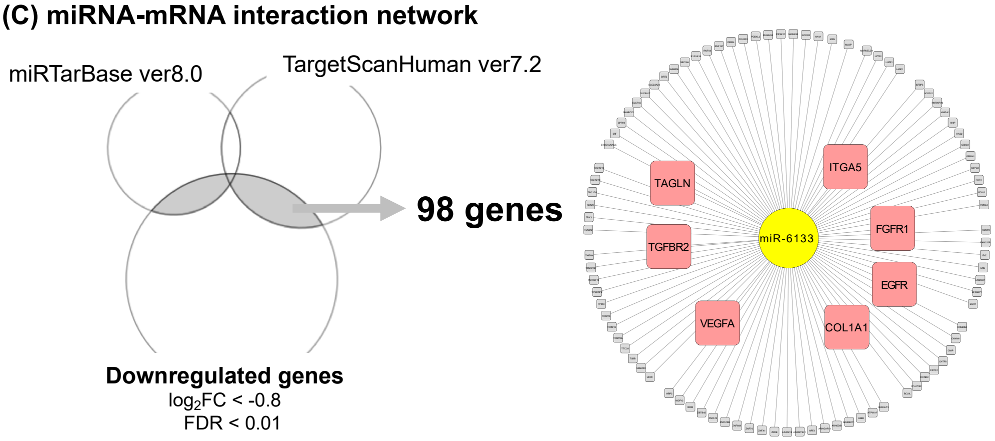

Figure 1. Characterization of genes controlled by miR-6133-5p. (A) Gene expression changes determined by RNAseq analysis were plotted according to fold change in log_2 (log_2 FC) and p-value in comparison with miR-6133-5p-treated and miControl-treated LX-2 cells. (B) A gene set enrichment analysis was performed using GSVA R package. The table showed the top downregulated enriched GO pathways. GSVA analysis indicated that the expressions of extracellular matrix–related genes were downregulated in miR-6133-5p-treated cells. (C) From the putative miR-6133-target genes predicted by TargetScanHuman version 7.2 and experimentally validated miR-6133-5p target genes listed in miRTarBase ver8.0 (http://mirtarbase.cuhk.edu.cn/php/index.php), we selected 98 genes downregulated in the miR-6133-5p-treated cells (fold change in log_2 ratio > 0.8, fold discovery rate <0.01, left panel). The genes annotated as 'extracellular matrix organization' are highlighted in a miRNA-mRNA interaction network generated using Cytoscape software (version 3.6.2).
